# Assessment toxic effects of exposure to 3-indoleacetic acid via hemato-biochemical, hormonal, and histopathological screening in rats

**DOI:** 10.1007/s11356-022-22026-8

**Published:** 2022-07-25

**Authors:** Hager Tarek H. Ismail

**Affiliations:** Department of Clinical Pathology, Faculty of Veterinary Medicine, Zagazig University, 1 Alzeraa Street, Zagazig City, 44511 Sharkia Province Egypt

**Keywords:** Indoleacetic acid, Hematology, Hepatorenal, Cardiac muscles, Testes, Histopathology

## Abstract

The current study purposed to investigate the 3-indoleacetic acid (IAA) possible adverse impacts on hematological parameters, hepatorenal function, cardiac, and skeletal muscles as well as testes of rats and histopathological alterations of respective organs and to determine the extent of reversing any adverse impacts occurred in animals after IAA withdrawal. Rats were exposed orally to 500 mg/kg BW by gastric intubation once daily for 14 days, after which one-half was sacrificed and the remaining half left for a further 14 days without IAA exposure. The exposure of rats to IAA produced anemia, leukopenia, neutrophilia, lymphopenia, and a significant increase in activities of serum transaminase, gamma-glutamyl transferase, creatine kinase-myocardial band, creatine kinase-muscle type, and levels of serum creatinine, sodium, chloride, and potassium. Furthermore, serum levels of testosterone, gonadotropins, and leptin significantly declined. The changes in most of measured parameters continued after IAA withdrawal. Histopathological alterations in different tissues supported these changes. In conclusion, subacute exposure to IAA at a high concentration could exert hematotoxicity and toxic effects on many soft organs and its withdrawal led to incomplete recovery of animals. Thus, IAA should be used cautiously as extensive use of it at high concentrations can cause harmful effects on the environment, animals and human beings.

## Introduction

Various plant growth regulators (PGRs), both natural or synthetic, are used nowadays in agriculture to improve the quality of crops and increase their resistance to diseases and harmful insects as well as to enhance their storage ability (Yeşilkaya et al. 2009). Plant growth regulators have five major groups recognized namely as auxins, cytokinin, gibberellins, ethylene generators, and abscisic acid. Auxins and cytokinins are considered the most important regulating growth and morphogenesis in plant tissue between other groups and are also available for use as exogenous synthetic regulants (George et al. 2008).

3-Indoleacetic acid (IAA) is the most abundant natural plant hormone of the auxin class. It controls many important physiological processes in the plants and considers the main known member of the auxins (Jatav et al. 2017). It can be produced by microorganisms also, such as bacteria and fungi (Fu et al. 2015), and its biosynthetic pathways in plants and bacteria are mainly similar, where both tryptophan (Trp)-dependent and Trp-independent IAA biosynthetic pathways occur in both of them (Mano and Nemoto 2012). The IAA molecular formula is C_10_H_9_ NO_2_, and its chemical structure is a monocarboxylic acid and one of the methyl hydrogens has been replaced by a 1H-indol-3-yl group (Giri et al. 2020). It is used commercially in wide forms, either dispersed in talc or in concentrated liquid compounds that can be diluted with water to induce more rapid plant growth, delay fruit drop, enhance root formation, and produce seedless varieties by parthenogenetic fruiting (Khan et al. 2007; Serrani et al. 2008; Moustakime et al. 2017). A foliar application of IAA has been used to increase fruit size with consequent increase in seed yield in various crops like groundnut (Lee 1990). Also, according to Reena et al. (1999) and Ashraf et al. (2006), IAA increased the seed yield of rice, sesame, and soybeans and IAA was successfully used to enhance the growth and yield of barley cultivars.

In spite of the importance of PGRs in agricultural fields, commercially used exogenous PGRs and those of natural origin produced by plants can accumulate in the environment and have different hazardous impacts on all living creatures if applied at inappropriate concentrations and times (Yeşilkaya et al. 2009). Also, anthropogenic activities are important way for the more accumulation of IAA in the environment (Barden, 2010) as well as the IAA degrades into hazardous products of carbon monoxide and oxides of nitrogen in the environment (Paranjape et al. 2015). Living organisms may be exposed to PGRs, including IAA via inhalation, dermal contact, or ingestion of contaminated water or food rich in vegetable stems (Yeşilkaya et al. 2009; Hac-Wydro and Flasiński 2015).

Although some experiments were performed on animals or cell cultures in vitro for assessment of the PGRs’ harmful impacts on animal and human (Folkes et al. 1999; Celik et al. 2006a), knowledge regarding the toxic effects of PGRs on mammalian organisms is limited or incomplete (Ozok and Celik 2012). Previous studies showed some effects of IAA on biological systems, such as inducing severe cytotoxicity via oxidative stress on lipid membranes and nucleic acids and increasing ROS generation (Folkes and Wardman 2001; de Melo et al. 2004). Also, according to Wardman (2002), IAA can boost apoptosis, necrosis, chromatin condensation, and DNA fragmentation.

On the other hand, there are also studies on IAA toxicity. John et al. (1979) observed that IAA possesses teratogenic effects in gestation mice and rats. Celik et al. (2002b) reported that the activities of aspartate aminotransferase, creatine phosphokinase, and lactate dehydrogenase were significantly increased by IAA after subacute exposure with 100 ppm dosages. Also, Furukawa et al. (2004) indicated that IAA induces neuronal apoptosis in the S phase and leads to microencephaly. Due the limitations of these previous studies, further studies should be directed to detect other possible IAA-induced toxicities. Therefore, the current study purposed to investigate the IAA possible adverse effects on erythrocytes, leukocytes, liver, kidneys, cardiac and skeletal muscles, and testes of adult albino rats as well as histopathological alterations of respective organs and to determine the extent of reversing any adverse impacts occurred in animals after IAA withdrawal.

## Materials and methods

### Tested agents

IAA (CAS No. 87–51-4) was purchased from Sigma-Aldrich Chemie GmbH (Germany). It was in the form of white to beige powder with purity ≥ 98% and was stored in a suitable condition. The virgin olive oil was obtained from an agricultural research center in Giza, Egypt.

### Animals

Adult male Wistar albino rats (*Rattus norvegicus*) were randomly selected (weighing 170 g ± 5). Rats (*n* = 36) were obtained from the laboratory animal house, Faculty of Veterinary Medicine, Zagazig University, Egypt, which were housed in standard cages and adapted for 7 days before starting the experiment. Animals were given standard feed and allowed to drink water ad libitum and were maintained in standard conditions at 25 ± 2 °C and relative humidity 45–60% in a light/dark cycle of 12/12 h. The health condition of experimental rats was assessed daily.

### Experimental procedure

Thirty-six rats were divided randomly into three groups, with 12 rats in each group as follows:

- Group I (control): rats were given only standard feed and water.
- Group II (vehicle): rats orally received 0.5 ml olive oil orally by gastric intubation once daily for 14 days.
- Group III (IAA): rats orally received IAA powder suspended in olive oil at concentration of 500 mg/kg BW by gastric intubation once daily for 14 days. The dose of IAA was selected based on the previously published studies (Furukawa et al. 2004). Bearing that in mind, the median lethal dose (LD_50_) of IAA in rats (oral treatment) is more than 500 mg/kg BW (Paley 2021). After 14 days of IAA exposure, the rats were maintained for another 14 consecutive days without any treatment.

### Sampling

Sample collection during the experiment course occurred twice, after 14 days from exposure to IAA and after 14 days of stopping exposure to IAA from the different experimental groups. Blood samples were collected from overnight fasted rats after being anesthetized by sodium pentobarbital by puncturing the retro-orbital venous sinus. The first part of blood specimens (1 ml) was collected in clean Wasserman tubes containing dipotassium salt of ethylenediamine tetraacetic acid (EDTA) for performing various hematological tests. The second part of blood specimens (1.5 ml) was collected in ordinary tubes and left to coagulate for centrifugation and separation of serum for performing the different biochemistry and hormonal assays. Rats were euthanized by decapitation after being anesthetized and the liver, kidneys, heart, skeletal muscle (extensor digitorum longus), and testes were excised quickly for histopathological studies.

### Hematological, clinical biochemistry, and hormonal assays

Hematological investigations included red blood cells (RBCs) count, hemoglobin (Hb) concentration, hematocrit (Ht) value, values of mean corpuscular volume (MCV), mean corpuscular hemoglobin (MCH), and mean corpuscular hemoglobin concentration (MCHC), and total and differential white blood cells counts were estimated using an automated blood cell counter (Sysmex XT-2000iV, Kobe, Japan; Buttarello and Plebani 2008).

Serum biochemistry parameters, including activities of alanine aminotransferase (ALT), aspartate transaminase (AST), gamma-glutamyl transferase (GGT), and creatine kinase-myocardial band (CK-MB) and levels of creatinine and urea were measured according to the manufacturer instructions by using available diagnostic kits purchased from Spinreact (Spain) and measured using the semi-auto chemistry analyzer (Chem-7 manufactured by Erba Diagnostics, Germany). Also, serum sodium (Na), chloride (Cl), and potassium (K) levels were measured using available diagnostic kits purchased from Spinreact (Spain), according to the manufacturer instructions and measured using of ST-200 Pro Electrolyte Analyzer (Sensa Core, India).

The measurement of serum creatine kinase-muscle type (CK-MM) occurred according to the kit guidelines by using an available ELISA kit (Catalogue No. RTFI00198) purchased from Assay Genie and by using the RT-2100C microplate reader (Rayto Life and Analytical Sciences, China).

The concentrations of serum hormone parameters, including luteinizing hormone (LH) (Catalogue No. CSB E12654r), follicle-stimulating hormone (FSH) (Catalogue No. CSB-E06869r), and leptin (Catalogue No. CSB-E07433r), were assayed using ELISA kits purchased from Cusabio according to the kit guidelines. Also, serum testosterone (Catalogue No. RTC001R) was measured using of ELISA kit purchased from BioVendor according to the kit guidelines. The measurement of serum hormone parameters occurred by using the RT-2100C microplate reader (Rayto Life and Analytical Sciences, China).

### Histopathological studies and lesion scoring

The liver, kidneys, heart, skeletal muscle (extensor digitorum longus), and testes were dissected, and specimens from fresh tissues were fixed in 10% formalin solution. After the fixation process is completed, the fixed tissues were dehydrated in ascending grades of ethanol, and then, the tissues were cleared in xylene and impregnated in paraffin wax. After that, they were sectioned at 5 µm using microtome and then stained with hematoxylin and eosin. The stained sections were viewed and photographed under a light microscope to detect the histopathological findings (Suvarna et al. 2013). A semiquantitative lesion scoring was estimated to express the degree of severity of the various histopathological changes observed in examined tissues (Gibson-Corley et al. 2013). According to the prevalence and severity of the histopathological changes in different examined tissues, the following scores were used: (- = no detectable histopathological lesion, +  = minimal or focal, +  +  = multifocal, +  +  +  = patchy or diffuse).

### Estimation of the IAA concentration in serum

#### Preparation of working standard and samples

The standard IAA was used to prepare a standard curve for quantitative comparison. 3-Indoleacetic acid standard (10 mg) was weighted in volumetric flask (10 ml) in methanol as diluent and was diluted to 50, 100, 500 ng/ml and 10, 50, 100 μg/ml in mobile phase then filtered by 0.22 μm syringe filter. Serum samples (200 μl/sample) were taken then added to methanol (500 μl) and vortexed for 2 min following by centrifugation at 10,000 rpm and filtrated by 0.22 μm syringe filter then finally collected in HPLC vial.

#### Chromatographic equipment and conditions

The HPLC–UV system consisted of Waters Alliance 2695 high-performance liquid chromatography (HPLC; Waters Alliance Instruments, Milford, USA) equipped with photodiode array detector (model 2996), low-pressure mixing system pump, vacuum degasser, auto-sampler with a sample loop of 100 µl, variable wavelength detector, and column Kromasil C18 (150 × 4.6 mm; 5 μm) (Nouryon, Sweden). Data processing was performed with using Empower Software. The IAA in the samples detected by HPLC under the following conditions: Flow rate 1/min with isocratic elution, the injection volume was 50 μl of sample, and the detection wavelength was set at 254 nm. Mobile phase: 0.2% glacial acetic acid: methanol 50%:50%, retention time is 4.6 min and run time is 7 min (Giri et al. 2020). Laboratory reagents were of analytical and HPLC grade and purchased from Sigma-Aldrich Inc. (St. Louis, MO, USA). Data were expressed as (μg/ml).

### Statistical analysis of data

The results obtained in this study were evaluated using SPSS software version 21 (IBM Corporation, Armonk, NY, USA) and were represented as mean ± SE. One-way analysis of variance (ANOVA) test and Tukey’s HSD post hoc descriptive were conducted to test the significance differences between the mean values except the concentration of IAA in the serum of group III after exposure and subsequent withdrawal of IAA exposure was tabulated and statistically analyzed by “*t*” test. The results of *p* < 0.05 were considered statistically significant (Snedecor and Cochran 1994) and were graphically drawn using GraphPad Prism 8 (GraphPad Software, San Diego, CA, USA).

## Results

### Hematological assay

**Fig. 1:**
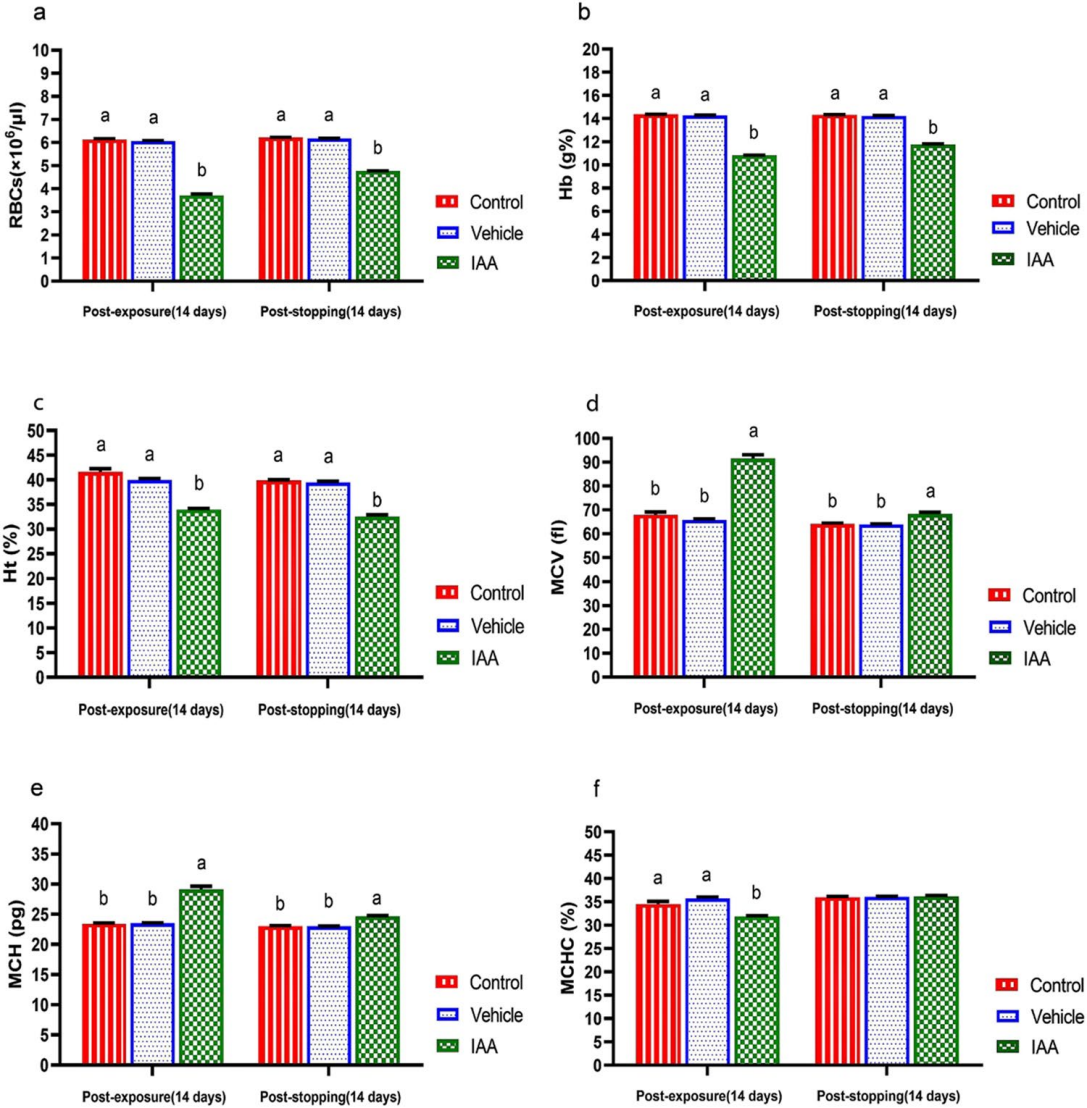
Effect of exposure and subsequent withdrawal of IAA exposure on erythrogram of experimental animals. Data were presented in the form of mean ± SE. Means bearing different alphabets within the same row are significantly (*p* < 0.05) different. No letters indicate (*p* > 0.05). IAA 3-indoleacetic acid, RBCs red blood cells, Hb hemoglobin, Ht hematocrit, MCV mean corpuscular volume, MCH mean corpuscular hemoglobin, MCHC mean corpuscular hemoglobin concentration

The exposure of animals to IAA caused a significant (*p* < 0.0001) reduction in RBCs count, Hb concentration and Ht value compared with the control group (Fig. 1a–c). These changes were also observed after stopping the exposure of rats to IAA. MCV and MCH values showed a significant (*p* < 0.0001) increase, while MCHC value showed significant decrease post-exposure to IAA in comparison to the control group (Fig. 1d–f). Stopping exposure of animals to IAA induced a significant (*p* < 0.0001) increase in the values of MCV and MCH and non-significant changes (*p* = 0.826) in the value of MCHC compared to the control group.

**Fig. 2:**
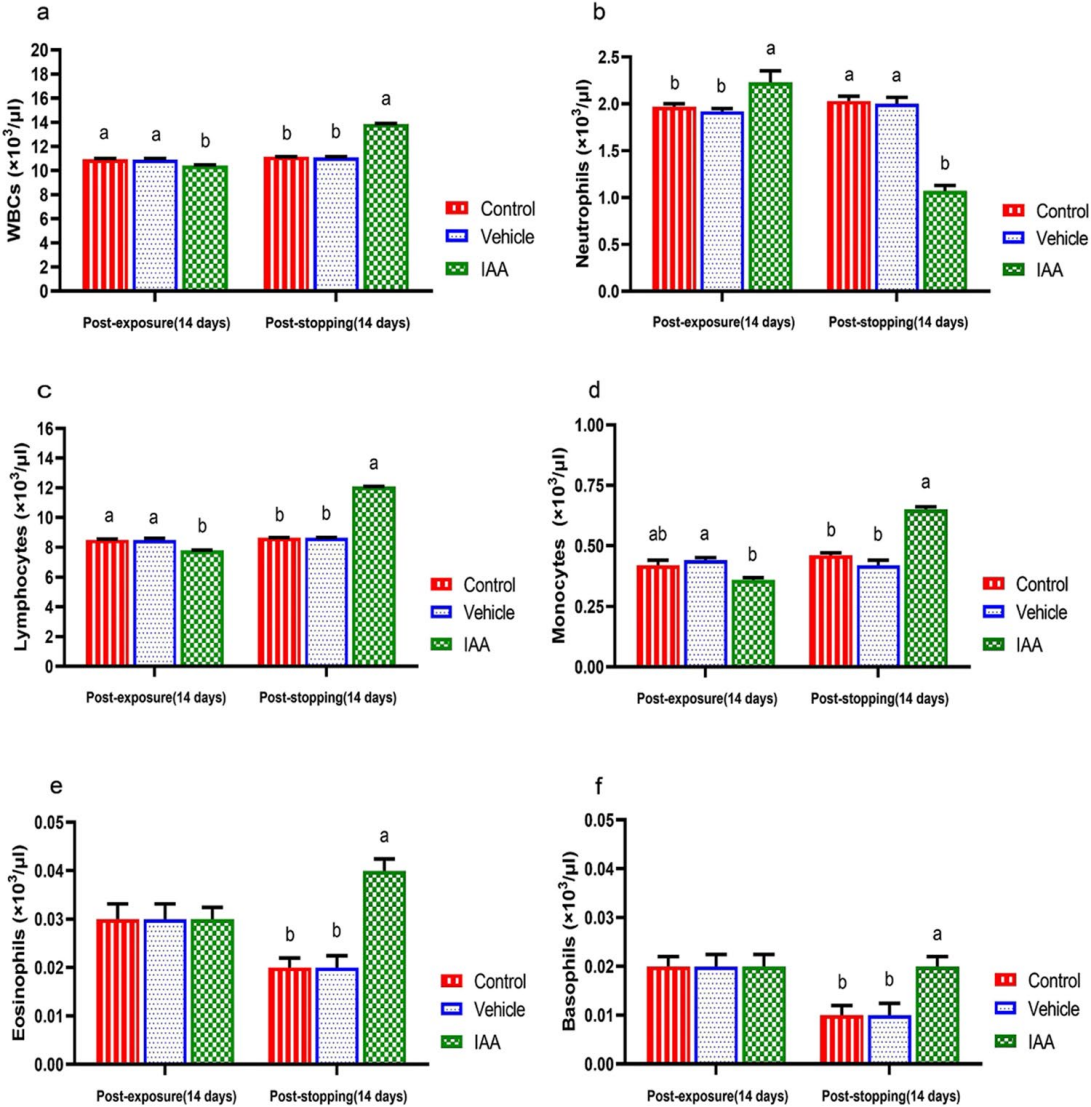
Effect of exposure and subsequent withdrawal of IAA exposure on leukogram of experimental animals. Data were presented in the form of mean ± SE. Means bearing different alphabets within the same row are significantly (*p* < 0.05) different. No letters indicate (*p* > 0.05). IAA 3-indoleacetic acid, WBCs white blood cells

As shown in Fig. 2a–f, leukopenia, neutrophilia, and lymphopenia (*p* < 0.0001) with an insignificant decrease in monocytes (*p* = 0.030) and non-significant change in eosinophils (*p* = 0.288) and basophils (*p* = 0.493) were observed in rats after exposure to IAA compared with the control group. Leukocytosis, neutropenia, lymphocytosis, monocytosis, eosinophilia, and basophilia (*p* < 0.0001) were observed after stopping exposure to IAA compared to the control group. No significant differences were recorded in all these hematological parameters in the vehicle group compared to the control group.

### Clinical biochemistry assay

**Fig. 3:**
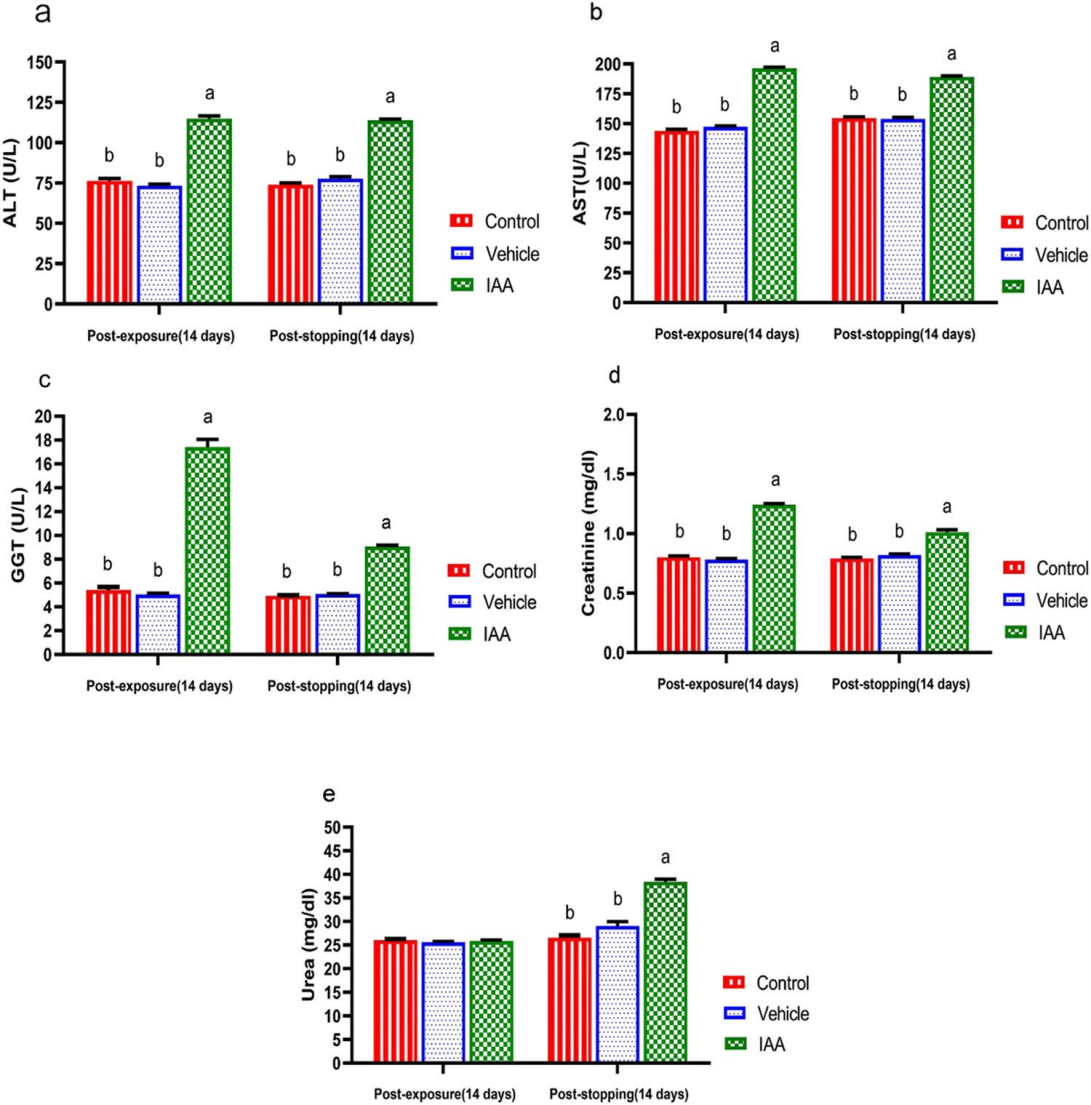
Effect of exposure and subsequent withdrawal of IAA exposure on some hepatorenal function markers in serum of experimental animals. Data were presented in the form of mean ± SE. Means bearing different alphabets within the same row are significantly (*p* < 0.05) different. No letters indicate (*p* > 0.05). IAA 3-indoleacetic acid, ALT alanine aminotransferase, AST aspartate aminotransferase, GGT gamma-glutamyl transferase

The effect of IAA on several serum hepato-renal markers in experimental rats is shown in Fig. 3a–e. Serum ALT, AST, and GGT activities and creatinine level were significantly higher than those in the control group (*p* < 0.0001). These changes were also observed in rats after stopping the exposure to IAA. Serum urea showed a non-significant change (*p* = 0.443) and a significant increase (*p* < 0.0001) after exposure and withdrawal of IAA, respectively, in comparison to the control group.

**Fig. 4:**
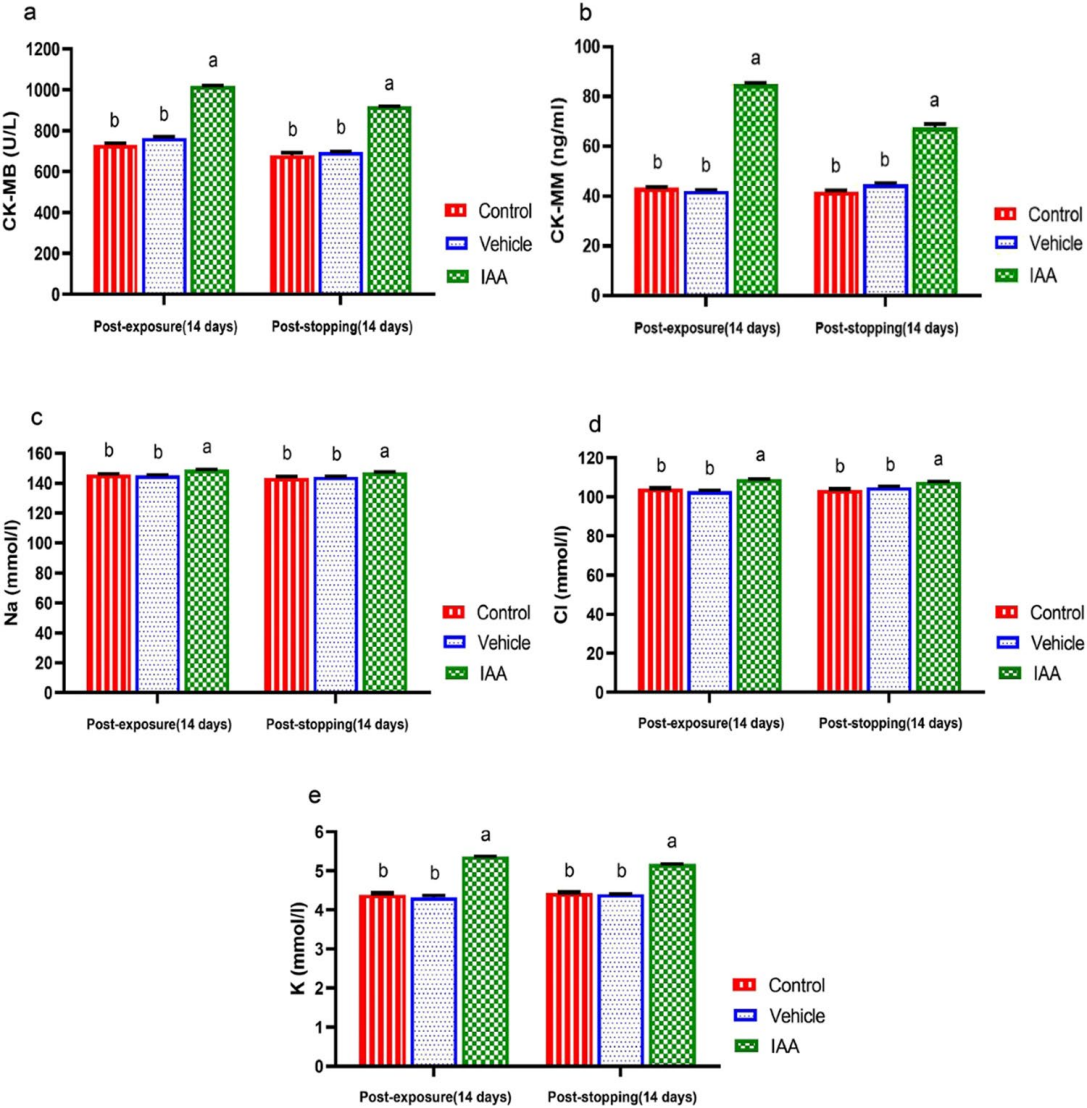
Effect of exposure and subsequent withdrawal of IAA exposure on CK-MB, CK-MM, and some electrolytes in serum of experimental animals. Data were presented in the form of mean ± SE. Means bearing different alphabets within the same row are significantly (*p* < 0.05) different. IAA 3-indoleacetic acid, CK-MB creatinine kinase-myocardial band, CK-MB creatinine kinase- muscle type, Na sodium, Cl chloride, K potassium

The variation in the serum activities of CK-MB and CK-MM and levels of Na, Cl, and K are shown in Fig. 4a–e. There was a significant increase in these parameters after exposure of animals to IAA and after stopping the exposure compared to the control group (*p* < 0.0001). No significant differences were recorded in all these parameters in the vehicle group compared to the control group.

### Hormonal assay

**Fig. 5:**
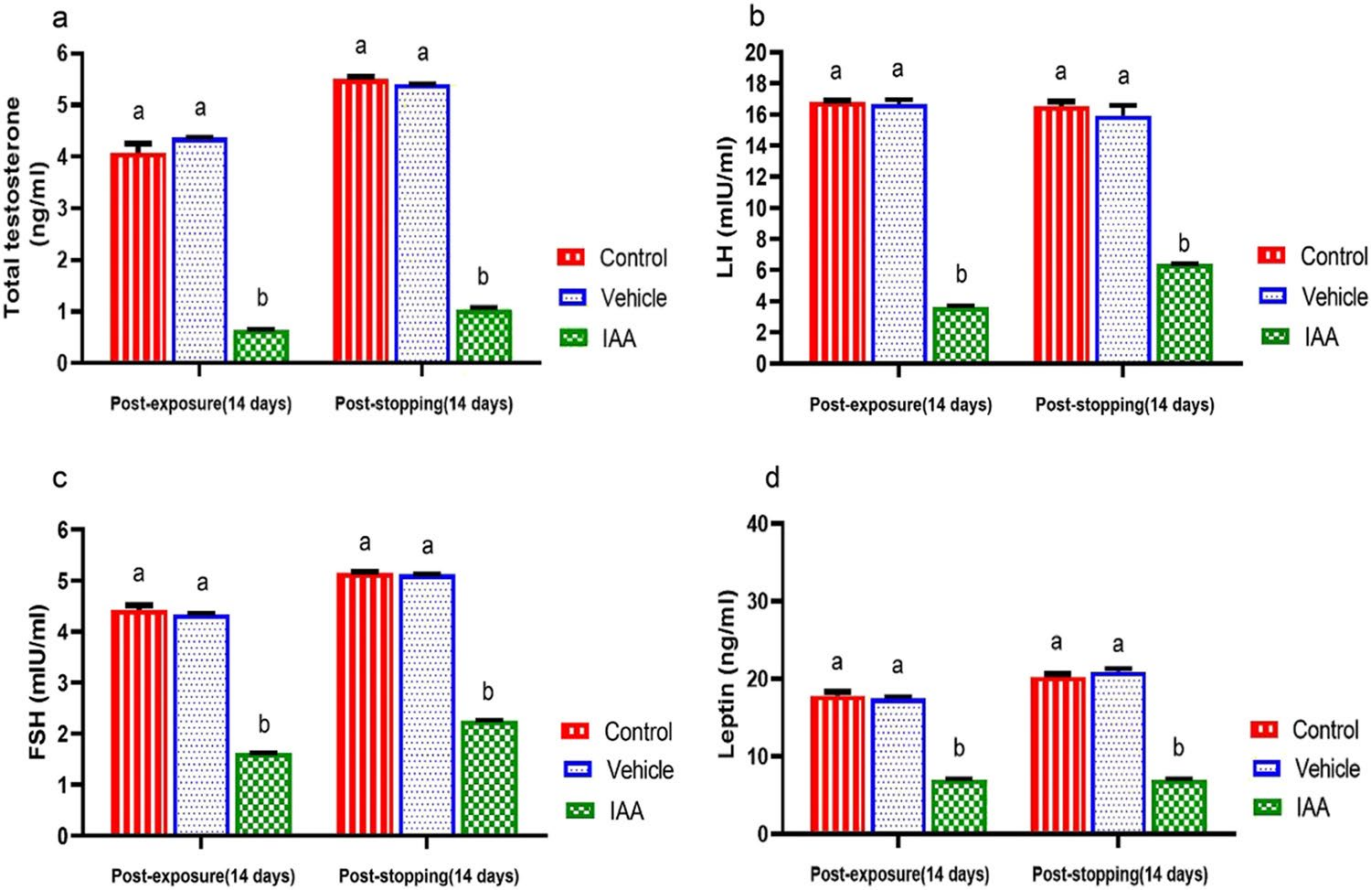
Effect of exposure and subsequent withdrawal of IAA exposure on serum total testosterone, gonadotropins and leptin of experimental animals. Data were presented in the form of mean ± SE. Means bearing different alphabets within the same row are significantly (*p* < 0.05) different. IAA 3-indoleacetic acid, LH luteinizing hormone, FSH follicle-stimulating hormone

Animals exposed to IAA elicited remarkable decrease (*p* < 0.0001) in the levels of serum total testosterone, LH, FSH, and leptin compared to the control group (Fig. 5a–d). These changes were also observed after stopping exposure of animals to IAA. The vehicle group showed non-significant changes in these parameters during the administration of olive oil and after stopping the administration.

### Histopathological changes

**Fig. 6:**
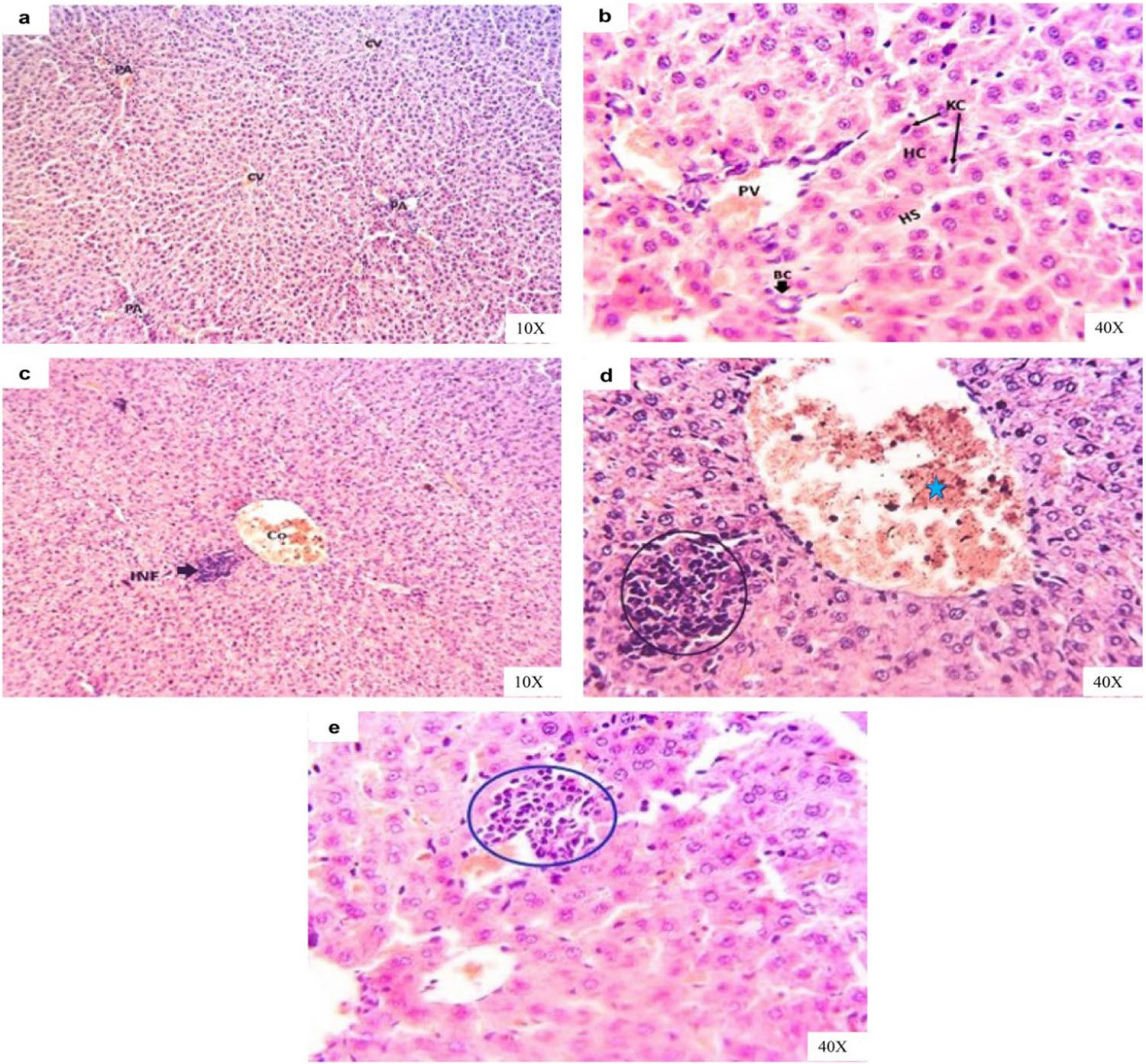
Representative photomicrographs of H&E-stained liver sections of rats in **a** control group showing normal hepatic tissue architecture, normal lobules with normal central vein (CV), and portal areas (PA). **b** Vehicle group showing normal hepatic cords (HC), hepatic sinusoids (HS), Kupffer cells (KC), portal vein (PV), and bile canaliculi (BC). **c** IAA-exposed group showing congested central vein (Co) followed by focal necrotic area replaced with inflammatory infiltrations (INF) and in **d** the high magnification of the previous image to show widening of central vein (star) associated with focal necrotic hepatocytes replaced with lymphocytes admixed with necrotic debris (circle). **e** IAA-withdrawal group still showing small focal inflammatory cells in the hepatic parenchyma (circle) with hypertrophied Kupffer cells

The examined hepatic sections of the normal and vehicle groups revealed normal hepatic architectures including lobules and portal areas (portal artery, vein, and bile ducts), which all lobules characterized by normal central vein, hepatic cells which arranged as a single or multiple cord interlacing with hepatic sinusoids and Kupffer cells during the experimental period (Fig. 6a, b). The liver sections from sacrificed rats exposed to IAA for 14 days displayed widening and congested central veins and other portal blood vessels associated with focal or/and multifocal necrotic areas mostly replaced with inflammatory cells infiltrations mainly lymphocytes admixed with necrotic debris (Fig. 6c, d). After IAA withdrawal for 14 days, the liver sections of experimental rats still revealed small focal inflammatory cells in the hepatic parenchyma with hypertrophied Kupffer cells and narrowing and sometimes absence of hepatic sinusoids due to swelling of hepatic cells (Fig. 6e).

**Fig. 7:**
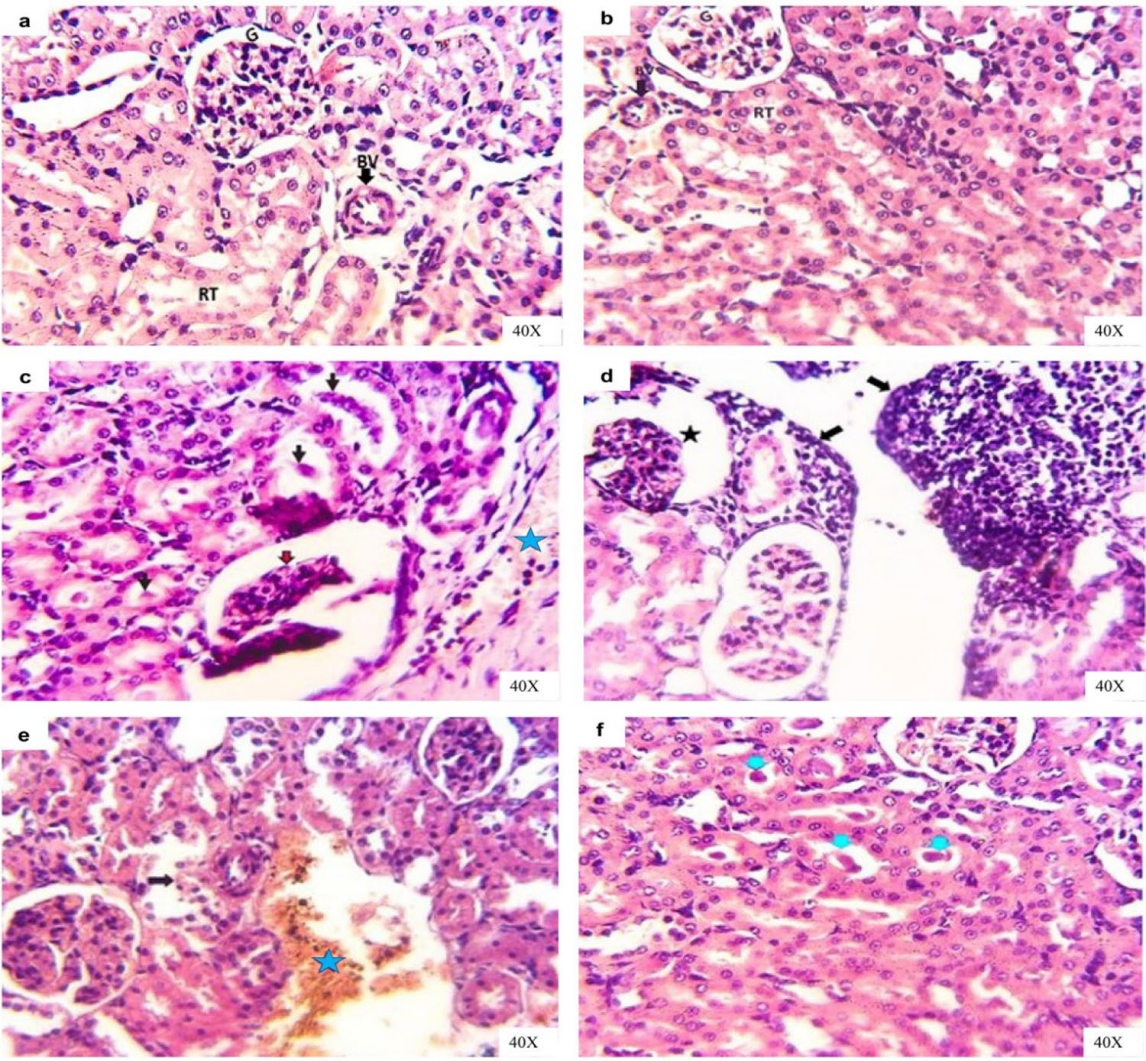
Representative photomicrographs of H&E-stained kidney sections of rats in **a** control group showing normal renal tissue architectures with normal glomeruli (G), normal renal tubules (RT), and normal renal blood vessels (BV). **b** Vehicle group showing normal renal tissue architectures with normal glomeruli (G), renal tubules (RT), and renal blood vessels (BV). **c** IAA-exposed group showing congested and pave mentation of inflammatory cells in large renal blood vessels (star) beside necrotic and shrunk glomeruli with large space and thickened glomerular capsule (red arrow), hyaline, and granular casts inside the renal tubules (black arrows) and in **d** showing massive inflammatory cells aggregations (arrows) and necrotic glomeruli (star). **e** IAA-withdrawal group showing nearly normal glomeruli followed with degenerated renal tubules (arrow) and interstitial hemorrhages (star) and in **f** showing hyaline castes inside renal tubules lumina (arrow)

Renal sections of the normal and vehicle groups revealed normal glomeruli (tufts, capsule and spaces), renal blood vessels, and proximal and distal renal tubules during the experimental period (Fig. 7a, b). Meanwhile, renal sections of IAA-exposed rats for 14 days suffered from congested large renal blood vessels that characterized with pave mentation of inflammatory cells (lymphocytes) beside necrotic and shrunk most glomeruli with large space and thickened of glomerular capsule; additionally, hyaline and granular casts inside the degenerated renal tubules were common (Fig. 7c). Other renal sections showed massive inflammatory cells aggregations (Fig. 7d). Renal sections of rats after IAA withdrawal for 14 days showed nearly normal glomeruli followed with degenerated renal tubules, interstitial hemorrhages, and hyaline casts inside renal tubules lumina (Fig. 6e, f).

**Fig. 8:**
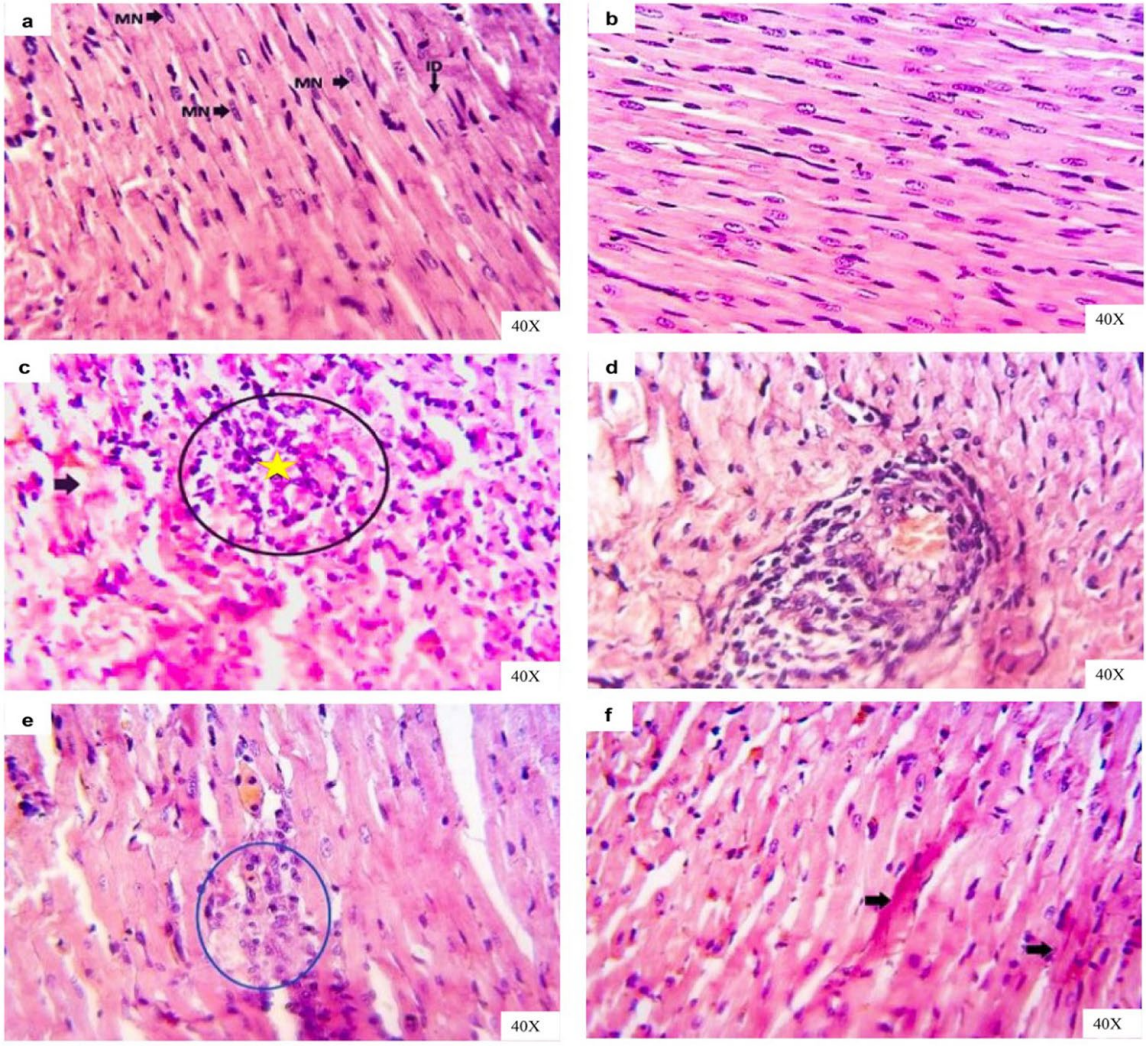
Representative photomicrographs of H&E-stained heart sections of rats in **a** control group showing normal cardiac muscle tissue architectures with arranged muscle striation fibers and normal cigar shaped nuclei (MN) and normal intercalated disc (ID). **b** Vehicle group showing normal cardiac muscle tissue architectures with arranged muscle fibers and inter-muscular spaces. **c** IAA-exposed group showing necrotic cardiac muscle fibers (circle), infiltrated with inflammatory cells (star) beside hyaline degenerations of the other fibers (arrow) and in **d** showing blood vascular cuffing by chronic inflammatory cells. **e** IAA-withdrawal group still showing small focal necrotic cardiac muscle (circle) and in **f** still showing hyalinized some cardiac muscle fibers (arrows)

Cardiac sections for both control and vehicle groups revealed normal arranged, striation fibers with normal cigar-shaped nucleus, and an intercalated disc during the experimental period (Fig. 8a, b). Moreover, cardiac muscle sections of the IAA-exposed rats for 14 days presented marked necrotic areas, which were usually replaced with inflammatory cells, as well as other fibers showed degeneration with loss of striations and became more eosinophilic (Fig. 8c). Other cardiac sections revealed perivascular cuffing (Fig. 8d). After IAA withdrawal for 14 days cardiac sections of rats still displayed small focal necrotic cardiac muscle with still hyalinized some fibers (Fig. 8e, f).

**Fig. 9:**
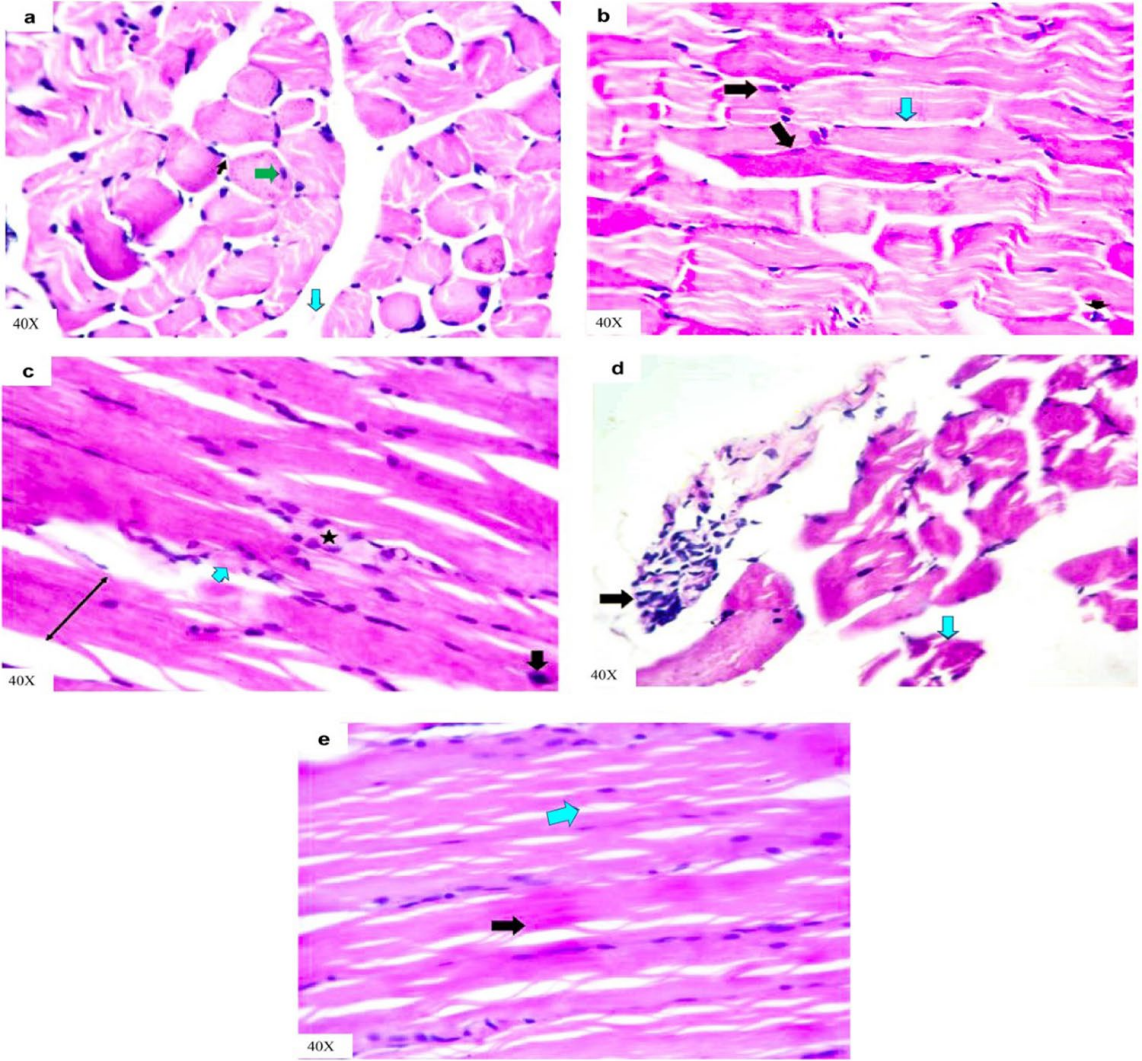
Representative photomicrographs of H&E-stained skeletal muscle cross and longitudinal sections of rats in **a** control group showing normal fasciculi each fasciculus separated from another by normal perimysium (light blue arrow); each fasciculus contains many muscle fibers with normal peripheral nuclei (green arrow) besides normal inter-fiber endomysium (thin black arrow). **b** Vehicle group showing normal non branched striations muscle fibers with normal peripheral nuclei (black arrows) besides normal inter-fiber endomysium (light blue arrow), which contains normal blood capillaries (arrowhead). **c** IAA-exposed group showing abnormal arranged muscle fibers represented by loss of inter-fibers spaces due to interstitial edema (two heads arrow), loss of striations (light blue arrow), and increase cellularity (star) besides dark apoptotic nuclei (black arrow) in **d** showing hyaline degenerated fibers (light blue arrow) and increase cellularity in epimysium area (black arrow). **e** IAA-withdrawal group still showing interfibrillar edema (light blue arrow) and remodeling of muscle fibers histological structures with normal striations (black arrow)

Skeletal muscle (extensor digitorum longus) sections for both control and vehicle groups revealed normal fasciculi each fasciculus separated from another by normal perimysium and contained many muscle fibers with normal peripheral nuclei besides normal inter-fiber endomysium, which contains normal blood capillaries along experimental period (Fig. 9a, b). Moreover, skeletal muscle sections of the IAA-exposed rats for 14 days presented abnormal arranged muscle fibers represented by loss of inter-fibers spaces due to interstitial edema, loss of striations and increase cellularity along with dark apoptotic nuclei (Fig. 9c). Other skeletal muscles sections revealed hyaline degenerated fibers and increased cellularity in epimysium area (Fig. 9d). After IAA withdrawal for 14 days skeletal muscle sections of rats still displayed interfibrillar edema and remodeling of muscle fibers histological structures with normal striations (Fig. 9e).

**Fig. 10:**
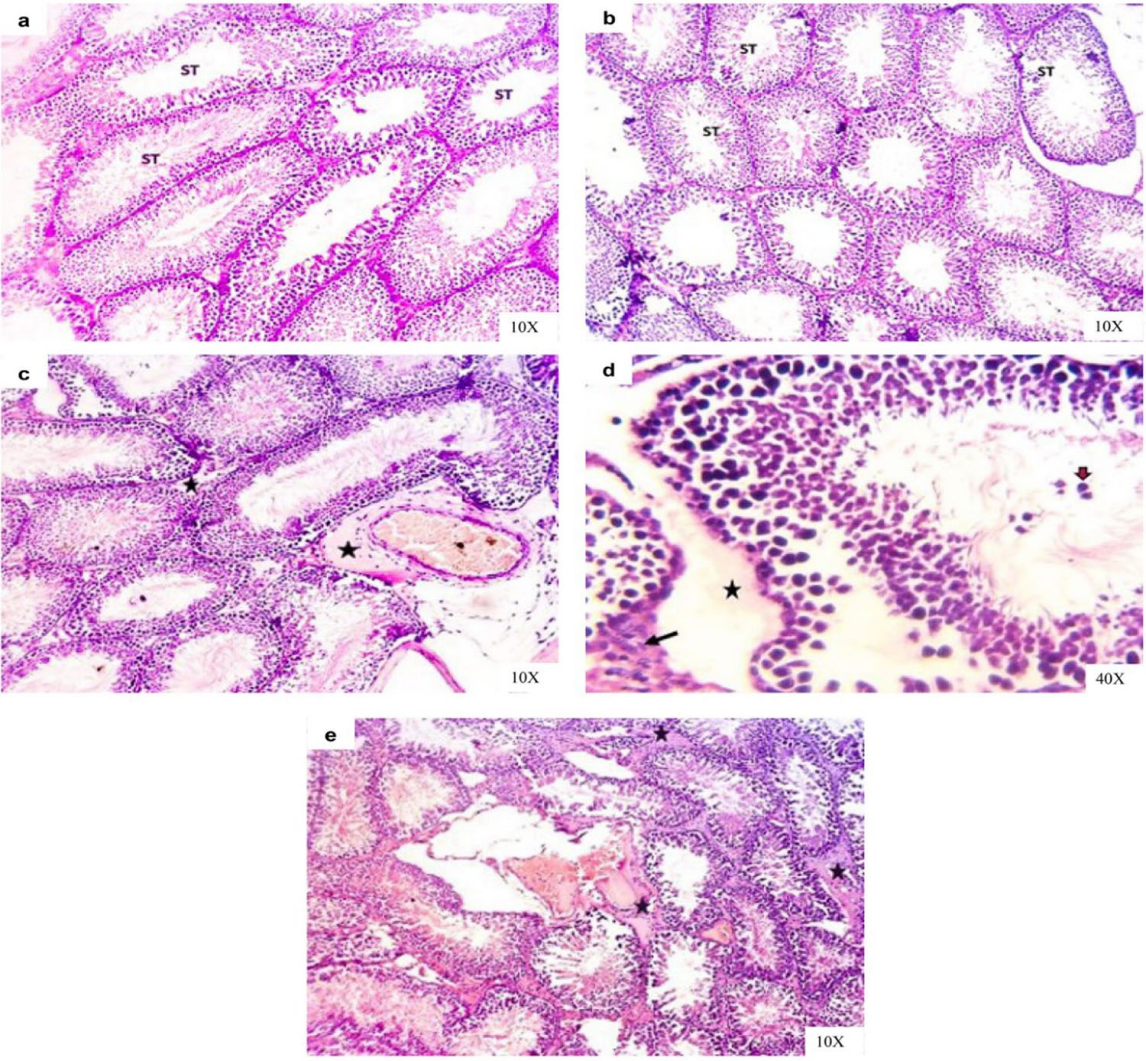
Representative photomicrographs of H&E-stained testis sections of rats in **a** control group showing normal testicular architecture with normal seminiferous tubules (ST). **b** Vehicle group showing normal testicular tissue architectures with normal seminiferous tubules (ST) and interstitial tissues. **c** IAA-exposed group showing thickened capsule followed by engorged of large blood vessels surrounded by inflammatory edema and interstitial between seminiferous tubules (stars) and in **d** showing high magnification of the previous section to show spermatogonia in the seminiferous tubule’s lumen (brown arrow), interstitial edema (star) and Leydig cells hyperplasia (black arrow). **e** IAA-withdrawal group showing interstitial and perivascular edema (stars)

Moreover, testis sections in both control and vehicle groups showed normal sized and histo-structures including seminiferous tubules, which contain all stages until reach spermatids and spermatozoa, interstitial tissues, and testis capsule during the experimental period (Fig. 10a, b). The examined testis sections of IAA-exposed rats for 14 days revealed a thickened capsule followed by engorged of large blood vessels surrounded by inflammatory edema in the interstitial between seminiferous tubules, seminiferous tubules degenerated with lumen spermatogonia and reduction of primary and secondary spermatocytes with abundant spermatids and spermatozoa (Fig. 10c, d). After withdrawal of IAA for 14 days, testis sections of rats still revealed interstitial edema, besides still decrease of spermatocytes and spermatids (Fig. 10e).

The different degrees of histopathological alteration and their distribution were observed in the different tissues in IAA-exposed rats for 14 days. At the same time, after IAA withdrawal for 14 days, the histological alteration severity in different tissues reduced, as shown in Table 1.

**Table 1:** Lesion scoring (semi-quantitative) in the liver, kidneys, heart, skeletal muscle, and testes tissues of rats after exposure and subsequent withdrawal of IAA exposure

Alteration criteria	Control group	Vehicle group	IAA-exposed group	IAA-withdrawal group
-Liver				
Kupffer cell hyperplasia	-	-	+	+ +
Congested blood vessels and sinusoids	-	-	+ + +	+
Inflammation	-	-	+ + +	-
Necrosis	-	-	+ + +	+
Degeneration	-	-	+ + +	+ +
- Kidneys				
Congested blood vessels	-	-	+ +	+
Inflammation	-	-	+ +	-
Necrosis	-	-	+ +	+
Degeneration/casts formation	-	-	+ + +	+ +
-Heart				
Interstitial edema/hemorrhage	-	-	+ +	-
Congested blood vessels	-	-	+ +	+
Inflammation	-	-	+ + +	-
Necrosis	-	-	+	-
Degeneration	-	-	+ + +	+ +
-Skeletal muscle				
Degeneration/loss of striations	-	-	+ + +	-
Interstitial edema	-	-	+ + +	+ +
Increase interstitial cellularity	-	-	+ + +	-
-Testes				
Interstitial edema/hemorrhage	-	-	+ +	+
Congested blood vessels	-	-	+ +	+
Inflammation	-	-	+ + +	+
Necrosis			+ +	+
Degeneration	-	-	+ + +	+

- = no detectable histopathological lesion, +  = minimal or focal, +  +  = multifocal, and +  +  +  = patchy or diffuse

### Serum IAA concentration

**Fig. 11:**
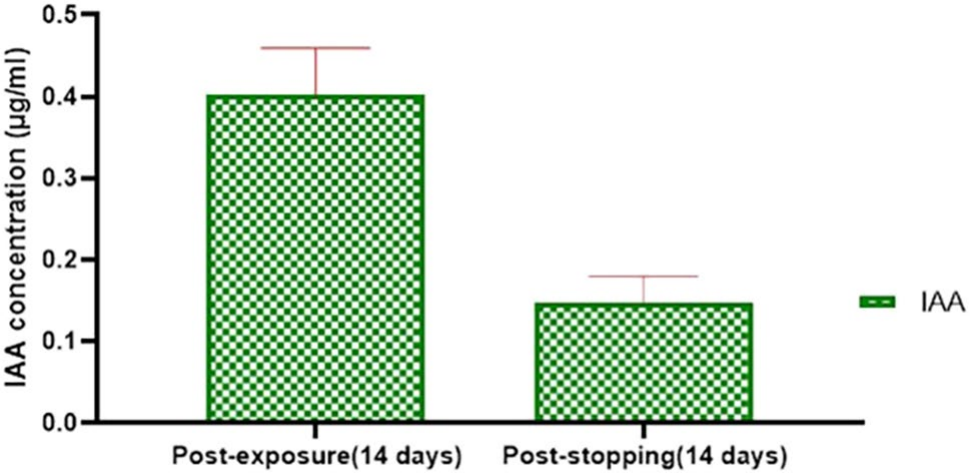
Serum concentration of IAA after exposure of rats for 14 days and subsequent withdrawal for 14 days in group III. Data were presented in the form of mean ± SE

As shown in Fig. 11, the serum concentration of IAA decreased significantly (*p* = 0.03) after stopping exposure of experimental animals in group III to IAA. The IAA was no detected in the serum of both the control and vehicle groups during the experiment.

## Discussion

The correct use of plant growth regulators leads to growing plants with high yield and free from diseases and subsequently the production of high-quality foodstuff at low cost. However, improper and unconscious use of plant growth regulators can have harmful effects on the environment and living creatures, where regulators can mix with the soil during agricultural applications and reach groundwater with infiltration. Also, regulators may have accumulated toxic effects in living bodies via the food chain (Sezgin and Kahya 2018).

Evaluation of hematological parameters can be a diagnostic tool for the adverse impacts of xenobiotics on the blood components of an animal. The exposure to chemical compounds at toxic doses frequently leads to alterations in several blood parameters are signal to hematological disorders (Arika et al. 2016). In this study, the exposure of experimental rats to IAA resulted a significant decline in RBC count, Hb concentration, and Ht value, which indicate anemia. Moreover, values of MCV and MCHC significantly increased and decreased, respectively, which indicate a macrocytic hypochromic anemia in the IAA-exposed rats. These hematological changes could be attributed to increasing the erythrocytes breakdown by exposure to IAA (Gupta 2019). IAA is one of the indole derivatives, which has an indole skeleton and indole possess hemolytic properties, where it has lipophilic characteristics and interacts with erythrocytes membrane (Weiss and Wardrop 2010). On the other hand, after IAA withdrawal, anemia was still present in experimental animals, but the value of MCV significantly increased and MCHC showed a non-significant change that indicates macrocytic normochromic anemia. IAA is shown to inhibit vitamin B12-dependent microorganism growth, so can cause macrocytic normochromic anemia due to Vit B12 deficiency (Rea and Patel 2018) and these explain the occurred changes in the erythrogram.

The current results revealed that IAA caused leukopenia associated with neutrophilia, lymphopenia and insignificant decrease in monocytes in experimental animals. These alterations may be due to a stress-induced diminishing number of the circulating lymphocytes, where the glucocorticoid-induce alterations in the “trafficking,” or lymphocyte redistribution from the blood to different body compartments. Also, circulating lymphocytes adhere to the endothelial cell layer that line blood vessels walls, and therefore, they were subjected to transmigration from the circulation into different tissues (such as lymph nodes, bone marrow and the spleen) where they are sequestered with a significant reduction in their circulating numbers (Cotter 2001). In contrast, glucocorticoids stimulate the inflow of neutrophils into the blood from bone marrow and attenuate the going out the neutrophils from the blood to body compartments (Cotter 2001). These induced alterations in leukogram are thought to make certain types of cells are routed to where they are needed during the stress response. Overall, one can say that stress exposure to xenobiotics induces a reduction in the count of lymphocytes and monocytes and increases the count of neutrophils (Marshall et al. 2014).

On the other hand, leukocytosis in experimental rats after subsequent IAA withdrawal may be associated with lymphocytosis, monocytosis, eosinophilia, and basophilia. These changes in leukogram may relate to acute inflammatory condition in the body, which may induce the increase in blood lymphocyte number in rats along with increasing the monocytes, eosinophils, and basophils number (Stockham and Scott 2008; Weiss and Wardrop 2010)‏, while the neutropenia in this condition arises from emigration neutrophils to tissues that exceed the bone marrow capacity to release neutrophils. Proinflammatory mediators and chemoattractants act on stimulate an increase in neutrophil margination besides firm adhesion to endothelial cells and tissue emigration, which cause the neutrophils circulating half-lives to be shorter and lowering the circulating neutrophils’ pool (Carter 2018).

The liver is the primary organ where foreign chemicals are metabolized and excreted. Consequently, hepatocytes are exposed to enormous concentrations of these chemicals, which can cause hepatocellular harm and hepatic dysfunction (Jaeschke 2015). The results of the current study demonstrated that hepatobiliary injury in rats after exposure to IAA, which was evident from the increase in the activities of different hepatic enzymes, such as ALT and AST as well as GGT in serum. The augmented activities of serum transaminase are an indicator of hepatocellular damage, where these enzymes leak into the circulation after the hepatocytes injury or alterations in cell membrane permeability. While the increasing in serum GGT activity is an indicator of cholestasis or bile duct necrosis (Kurtz and Travlos 2017). Moreover, AST is present also in high concentrations in the kidneys, heart, and skeletal muscles, so injury in any of these tissues may rise the serum AST activity (Naik 2016). This data in agreement with the results of Celik et al. (2002b, a), who found that the activity of serum AST was increased significantly after subchronic exposure of rats to IAA and Abed El-Aliem and Ebrahem (2003), demonstrated that the exposure of rats to indole-3-butyric acid (IBA), the precursor of IAA, increased serum ALT and AST activities.

Toxic nephropathies are an influential and relatively common category of renal injury, which are defined mainly as renal injury caused by several medications, diagnostic substances and toxin exposures, which include environmental agents and chemicals (Perazella 2010). The findings of this study showed that rats exposed to IAA exhibit remarkable impairment in renal function, which was confirmed by the increase in serum creatinine level. This finding was in harmony with those reported by Niwa et al. (1994), who have demonstrated that the administration of indole increased serum creatinine concentration in rats; IAA is a derivative of indole, and Abed El-Aliem and Ebrahem (2003) reported that the exposure of rats to IBA increased serum creatinine level. On the other hand, the assessment of serum urea is less valuable than the serum creatinine level in diagnosis of renal function as the urea level in the serum is affected by many factors other than renal function. Serum urea concentration is changed according to rate of urea synthesis by the liver and rate of clearance by the kidney. According to that, the hepatic dysfunction decreases urea production, so in case of combined hepatic and renal dysfunction (as in hepatorenal syndrome), serum urea concentration can be normal but not due to normal renal excretory function (Pagana and Pagana 2013). This explains the normal serum urea level after exposure of rats to IAA. The obtained microscopic changes in hepatic and renal tissues confirmed the obtained biochemical findings in this study (Figs. 6 and 7).

Creatine kinase (CK) is a relatively muscle-specific enzyme that has different isoenzymes, which play a role in determining the different injured tissues in the body. From these isoenzymes, CK-MB, which predominates the cardiac muscle and CK-MM, is the most prevalent in the skeletal muscle (Kurtz and Travlos 2017). A significant elevation in the activity of serum CK-MB in IAA-exposed animals may be due to cardiac tissue injury, where CK-MB presents mainly in cardiac muscle cells and leakage to the circulation after myocardial cell injury or necrosis (Gupta 2012), while the increase in the activity of serum CK-MM indicates skeletal muscle cells damage (Chinoy and Cooper 2018). On other hand, myocyte injury may lead to electrolyte imbalance (Zhang 2012). According to the results of this study, the exposure of experimental rats to IAA leads to hypernatremia and hyperchloremia may be due to the shift of water from the extracellular to the intracellular compartment where the myocytes injury condition generates new osmoles in skeletal muscle cells (Halperin et al. 2010). Hyperkalemia may result from the release of large quantities of intracellular potassium from injured muscle cells to plasma (Kaneko et al. 2008). The histopathological alterations in cardiac and skeletal muscles confirmed these findings (Figs. 8 and 9).

The spermatogenesis process depends primarily on testosterone secretion and pituitary gonadotropins action, such as luteinizing hormone (LH), which stimulates testosterone production and secretion by Leydig cells and follicle stimulating hormone (FSH), which stimulates testicular growth and enhances the production of an androgen-binding protein by Sertoli cells (MacLachlan et al. 2002; Spaliviero et al. 2004). The results of the present study revealed that IAA-exposed animals showed low serum concentration of testosterone together with low serum FSH and LH levels. These findings suggested that IAA disturbs the production of sex hormones in male rats by induction of Leydig cell hyperplasia, and subsequently, the produced testosterone is rapidly aromatized to estradiol because of increased aromatase activity, and finally, one can observe the diminution in serum testosterone level (Hayes 2008). On the other hand, there is a decline in serum levels of gonadotropins (LH and FSH) due to negative feedback to the increase in estradiol level (Melmed and Conn 2005‏). These findings agreed with that of Hassan et al. (2013) who demonstrated that a significant decrease in serum testosterone level in mice after exposure to IAA and Abed El-Aliem and Ebrahem (2003) who reported that the exposure of rats to IBA decreased serum testosterone level.

Leptin not only plays significant role in regulating energy homeostasis but also takes part in the spectrum of serious physiological activities in the body, such as neuroendocrine and immune function, lipid, glucose and bone metabolism and reproduction (Park and Ahima 2015). Leptin regulates reproductive functions by changing the sensitivity of the pituitary gland to gonadotrophin-releasing hormone (GnRH). Also, it can act on all levels of the hypothalamus–pituitary–gonadal (HPG) axis where its receptors can be found in cells throughout the HPG axis and may have local effects on the function of testis and spermatogenesis (Zhang and Gong 2018). The results of the current study demonstrate that exposure of rats to IAA reduced serum leptin level may be due to the impact of possible stress on animals, which may attribute to the dose of tested agent and/or experimental procedures (Everds et al. 2013). According to Haleem 2014, an increase in the activity of the hypothalamus–pituitary–adrenal (HPA) axis during exposure to stressors would be associated with a corresponding decline in leptin release. After indicating a link between the leptin level and reproductive system, one can say, the reduction in circulating leptin level may be associated with a notable decrease in secretion of the gonadotropins (LH and FSH) and subsequently result in gonadal dysfunction (Cunningham et al. 1999). The obtained results concerning the hormonal changes were in relation with the obtained histopathological alterations in the testis (Fig. 10).

According to some previous studies, the injuries and alterations in the hepatorenal system, cardiac and skeletal muscles and reproductive system in response to IAA exposure could be explained by several reasons. According to Alanazi et al. (2021), IAA stimulates the generation of ROS and causes an increase in oxidative stress, which is associated with the loss of cell membrane integrity, DNA fragmentation and chromatin condensation. Also, cellular pH decreases due to increased ROS levels caused by IAA and subsequently the cells become semi-oxidized and thus, the potential for increasing apoptosis.

Also, according to pervious data by Celik et al. (2002b and 2006b), the subacute exposure to IAA caused a significant decrease in the activity of glutathione reductase (GR) in the liver and the superoxide dismutase (SOD) in the heart. While liver and kidney MDA levels were increased significantly by IAA exposure. On the other hand, according to Hassan et al. (2013), animals exposed to IAA experienced adverse effects on the testicular functions by increasing oxidative stress and inhibiting the endogenous antioxidant system. Moreover, according to Topalca et al. 2009, administrations of indole-3-butyric acid at subacute and subchronic levels cause changes in the antioxidant defense systems in various tissues (brain, kidney, heart, muscle, liver, lungs and spleen) of rats.

According to the data of this study, the concentration of IAA in the serum of rats after 14 days from stopping the exposure reduced in comparison to its concentration after the exposure time and these may lead to stepwise reduction in alterations in the measured parameters due to the diminishing of toxicant in a systemic circulation. On the other hand, incomplete recovery of animals after stopping the exposure may be due to slow clearance of IAA from circulation and/or slow or incomplete reversibility of adverse impacts besides the reduction in the rate of its elimination from the body due to renal dysfunction (Ashauer et al. 2010; Watanabe et al. 2021).

## Conclusions

Considering the outcome of current study, I concluded that the subacute exposure to IAA at a high concentration could exert hematotoxicity appeared in form of a decrease in erythrogram parameters and leukopenia as well as hepatorenal dysfunction and various toxic effects on heart and testis in addition to skeletal muscles. The alterations in the hemato-biochemical and hormonal tests as well as the histological structure of different organs revealed these toxic effects. Also, the withdrawal of IAA resulted in incomplete recovery of animals from adverse impacts within the time course of the experimental investigation, so the recovery from such impacts could need more time. Thus, IAA should be used cautionary as extensive use of it in high concentrations can cause harmful effects on the environment, animals and human beings.

## Data Availability

All datasets generated and/or analyzed during this study are included in this article.
